# Evaluation of radiofrequency electronic system in intraoperative monitoring of surgical textiles

**DOI:** 10.1590/S1679-45082018AO3997

**Published:** 2018-04-19

**Authors:** Adriana Marco Antonio, Carlos Andre Pereira Vieira

**Affiliations:** 1 Centro de Experimentação e Treinamento, Hospital Israelita Albert Einstein, São Paulo, SP, Brazil.

**Keywords:** Textiles, Foreign bodies, Radio waves, General surgery, Technology assessment, biomedical, Secondary prevention, Surgical sponges, Radio frequency identification device, Têxteis, Corpos estranhos, Ondas de rádio, Cirurgia geral, Avaliação da tecnologia biomédica, Prevenção secundária, Tampões de gaze cirúrgicos, Dispositivo de identificação por radiofrequência

## Abstract

**Objective:**

To test performance of SurgiSafe^®^, a radiofrequency electronic device to detect surgical textiles during operations as compared to manual counting.

**Methods:**

Surgical sponges with radiofrequency TAGs were placed in the abdominal cavity of a pig submitted to laparotomy, in randomly distributed sites. The TAGs were counted manually and also using SurgiSafe^®^. Positive and negative predictive values, sensitivity, specificity and time required for counting were analyzed for both methods.

**Results:**

Through the analysis of 35 surgical cycles, SurgiSafe^®^ immediately identified all sponges, with specificity, sensitivity, positive and negative predictive values of 100%. Although not statistically significant, the manual count had sensitivity of 99.72% and specificity of 99.90%.

**Conclusion:**

SurgiSafe^®^ proved to be an effective device to identify surgical sponges *in vivo,* in real time; and its use as an adjuvant to manual counting is very helpful to increase patient's safety.

## RESUMO

**Objetivo:**

Testar o desempenho do SurgiSafe^®^, dispositivo eletrônico de detecção de têxteis cirúrgicos por radiofrequência no intraoperatório, comparado à contagem manual.

**Métodos:**

Gazes com etiquetas de radiofrequência (TAGs) foram alocadas na cavidade abdominal de um suíno submetido à laparotomia, em locais distribuídos aleatoriamente. As TAGs foram contadas manualmente e com uso do SurgiSafe^®^. Valores preditivos positivos e negativos, sensibilidade, especificidade e tempo de contagem foram analisados para ambos os métodos.

**Resultados:**

Por meio da análise de 35 ciclos cirúrgicos, o SurgiSafe^®^ fez a identificação instantânea de todas as gazes, com especificidade, sensibilidade, valores preditivos negativo e positivo de 100%. Apesar de não apresentar significância estatística, a contagem manual apresentou sensibilidade de 99,72% e especificidade de 99,90%.

**Conclusão:**

O SurgiSafe^®^ mostrou-se eficaz para contabilização de têxteis cirúrgicos em tempo real *in vivo,* e seu uso como adjuvante na contagem manual é de grande valor para o aumento de segurança do paciente.

## INTRODUCTION

The inadvertent retention of foreign bodies (IRFB), a lawfully indefensible malpractice,^(^ ^1^ ^)^ is a problem that exists since humans began to perform surgical procedures,^(^ ^2^ ^)^ and it persists despite the established protocols.^(^ ^3^ ^,^ ^4^ ^)^ Reviewing the literature reveals a frequency of one inadvertent retention for every 7,000 surgeries,^(^ ^5^ ^)^ consisting mostly of textile foreign objects.^(^ ^6^ ^)^

In Brazil, 43% of surgeons left foreign bodies, and 73% of surgeons removed foreign bodies in the last years, and 90% of the foreign bodies were textile materials. The retention was disclosed to patients in 46% of cases, and of these, 26% sued the physicians or the organization.^(^ ^7^ ^)^

Inadvertent retention of foreign bodies can lead to death of the patient^(^ ^8^ ^)^ or leave several sequelae, such as otitis,^(^ ^9^ ^)^ infection or sepsis,^(^ ^10^ ^)^ visceral perforation,^(^ ^3^ ^)^ gossypiboma in the abdominal cavity^(^ ^11^ ^)^ and in various regions of the body.^(^ ^12^ ^-^ ^14^ ^)^

Predisposing factors for IRFB include risks associated with the process, and the most reported ones are changes in the surgical team during surgery, less compliance to pre- and post-operative counting, and inconsistent policies on the use of intraoperative images;^(^ ^3^ ^)^ risks associated with the procedure, such as urgency, multiplicity, complexity, and duration of surgery;^(^ ^3^ ^,^ ^8^ ^)^ and other risks, ranging from cases involving obese patients,^(^ ^8^ ^)^ blood loss, and the participation of resident physicians.^(^ ^10^ ^)^ Inadvertent retention of foreign bodies is strongly associated with incorrect counting during the procedure.^(^ ^5^ ^)^

The worldwide patient safety movement led the Brazilian government launch the National Program for Patient Safety, which established the Safe Surgery Protocol.^(^ ^15^ ^)^ However, checklist filling out is significantly incomplete; the surgical textile items count must be confirmed orally by the nursing team or the scrub nurse before the patient leaves the operating room,^(^ ^16^ ^)^ which means developing technology-based improvements is imperative.^(^ ^5^ ^)^

## OBJECTIVE

To compare SurgiSafe^®^ with the manual count procedure, in terms of sensitivity, specificity, counting time, and positive and negative predictive values.

## METHODS

The study was conducted at the Experiment and Training Center (CETEC - *Centro de Experimentação e Treinamento*) of *Hospital Israelita Albert Einstein* (HIAE) and approved by the Ethics Committee on Animal Use of HIAE under protocol 2248-14.

**Figure 1:**
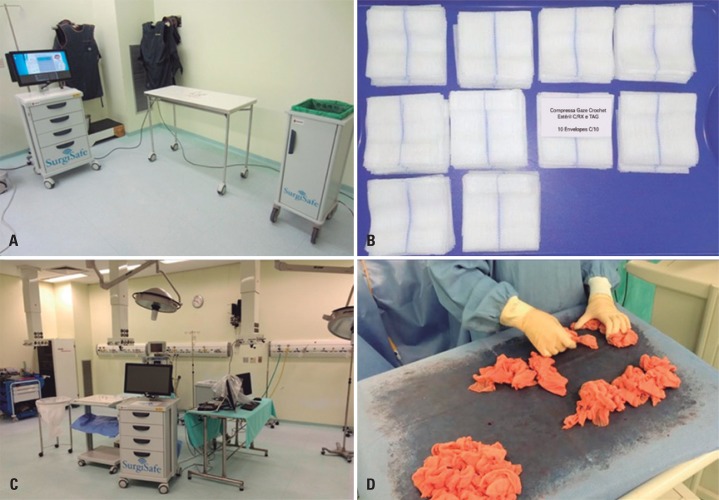
SurgiSafe^®^ apparatus. (A) SurgiSafe^®^ image, (B) tagged surgical sponges, (C) operating room, and (D) manual count of surgical textile items

The SurgiSafe^®^, a duly registered device (Figure 1) approved by the National Health Surveillance Agency (ANVISA - *Agência Nacional de Vigilância Sanitária*) for hospital use, is a system that counts surgical textile items using radiofrequency identification (RFID). It was designed by the company Target Empreendimento Ltda., for continuous audit of surgical textile items, and launched in November 2014.

More than just counting, the system integrates a management flowchart, in case of discrepancy. If a surgery is finished with a discrepancy, in addition to a report describing the serial number of each textile item used, notices are e-mailed to medical and nursing managers of the operating room, as well as to the clinical director of the hospital. From the institutional point of view, a sentinel event is created, and the patient is then monitored and not discharged until absence of IRFB is guaranteed.

The Ultra high frequency antenna used by the system generates a radio wave at a specific frequency, respecting local laws in Brazil, the approved frequencies determined by the National Telecommunications Agency (ANATEL – *Agência National de Telecomunicações*) are between 914 and 930mHz. This electromagnetic wave hits a small antenna connected to a chip. The design of the antenna took into consideration the blood and the inner parts of the human body, in order to optimize the tuning. When the antenna is struck by the electromagnetic wave, it generates an electric current that activates the chip. A reply is sent by this chip with its serial number and an electronic product code (EPC). The code enables identification of the chip's manufacturer, bar code and serial number. In this way, every textile item has a unique identity. In spite of the apparent complexity, the equipment screens only show the information needed for the surgical team and the circulating nurse, in different screens, with simple menus.

No interaction of SurgiSafe^®^ with the electrical medical equipment of the operating room was observed. For this purpose, we utilized a SurgiSafe^®^ prototype, with the same electronics, but without the proper shielding designed for the original equipment. During the procedure, we used the following equipment: a Fabius (Dräger) ventilator with its multiparameter monitor, a Valley Lab electrocautery, a Philips ultrasound equipment, and a manual external defibrillator. None of these devices showed abnormal behavior, and none of them interfered with SurgiSafe^®^, which did not stop the counting process during the entire procedure.

Gauze pads measuring 7.5cm x 7.5cm were used. Each gauze was identified by an extensive numerical sequence encoded on an identification chip responsive to a specific radiofrequency. This chip has an insulating sheath, which prevents contact with blood and is securely attached to the surgical textile item. The surgical textile items are connected by wires with proven efficiency and biocompatibility. A pad made of gauze involves the radiofrequency identification tag (RFID TAGs) with rounded edges to prevent it from causing lesions in viscera. The RFID TAGs consists of a chip attached to an antenna, wrapped in a polymer with proven biocompatibility. Benchtop tests proved successful for electrical and liquid insulation, and also for temperature of the scalpel tip and contamination of the operative site, with resistance to the routine use of the instruments.

To assess SurgiSafe^®^ *in vivo*, a randomized, doubleblind study was conducted, using a 35kg Yucatan/Minnesota pig. For the surgery, the pig was placed in the supine position, under general anesthesia. A median laparotomy with a zipper suture in the midline was performed to access the cavity. Gauze pads equipped with RFID TAGs were placed in the abdominal cavity, varying in number and location. An Excel spreadsheet generated a random number of pads, ranging from zero to four, for each of the main anatomical regions of the abdomen, grouped according to its topographical anatomy in nine regions: right and left hypochondrium, right and left flank, right and left iliac fossa, epigastrium, mesogastrium and hypogastrium. The surgeon had the gauze pads placed in number and location determined by the randomization and closed the cavity, approaching the edges of the walls by crossing the wires. He moved out of the operative field, and the observer and his assistant entered the room. The observer removed the gauze pads held by cotton threads and counted them manually. The time required for each count, monitored by two auxiliaries with digital stopwatches, was recorded. At the end of the counting, the observer left the room, and the surgeon returned and performed a new distribution of tags according to the randomization. These cycles were repeated one hundred times. For SurgiSafe^®^ counting, the gauze pads were placed on the auxiliary table in stacks to ensure readability. As the pads were disposed in the waste bin, their quantity was identified. The difference between the number of pads on the auxiliary table and in the bin was considered to be in the surgical field. Then, the system automatically generated a PDF report, with the serial numbers and quantity of tags, to which the observer did not have access.

Besides SurgiSafe^®^, which consists of a fixed treadmill type radiofrequency antenna (hamper), positioned under the auxiliary table and the waste bin in the operating room, two other types of portable radiofrequency antennas, with different designs, called “wand” and “far-field antenna” (FF antenna), were also tested. The observer scanned the pig with rotational movements of the FF antenna and the wand, respectively, to identify and quantify the tags in the abdominal cavity. At the end of the scan, the observer removed the gauzes and counted them manually.

The first 53 cycles were used for fine adjustment of SurgiSafe^®^, in order to neutralize any interference that could be generated. The next 12 cycles were used to change the positioning of the device's antenna. The purpose of these first 65 procedures was to adjust the parameter settings to be used as a rule when using SurgiSafe^®^ and did not need to be repeated at any time during the use of the apparatus. The remaining 35 cycles were performed for the purpose of statistical analysis, in the comparison of manual and SurgiSafe^®^ counting. The gauzes pads were the same, from the beginning to the end of the experiment, when they were tested and were working normally.

To compare the methods, we used the χ^2^ test at 5% probability. The sensitivity was calculated by the ratio of the total of correct positive results to the sum of correct positive and false-negative results. The specificity was calculated by the ratio of the total of correct negative results and the sum of correct negative and false-positive results. The positive predictive value was calculated by dividing the number of true positive results by the sum of true and false-positive results. The negative predictive value was calculated by dividing the number of true positive results by the sum of true and false negative results.

Sensitivity, specificity, and predictive values were analyzed for SurgiSafe^®^ and manual counting, considering 35 homogeneous cycles of repetitions, under the same experimental conditions, performed by a single professional, during a 5-hour period, with a total of 1,070 textile items.

## RESULTS

Although no significant differences were found between manual count and SurgiSafe^®^ counting (χ2=0.13; p>0.98) in terms of sensitivity and specificity, the manual count value differed from the correct value in four instances - with lower values in three instances, and a higher value in one - considering the absolute values. Both positive and negative predictive values were 100% (Table 1).

**Table 1:** Comparing results of SurgiSafe^®^ count (immediate counting) and manual count

Cycle number	Total number of gauzes	Total SurgiSafe^®^ on table	Total manual count	Manual count time
1	23	23	23	44
2	26	26	26	52
3	30	30	30	72
4	22	22	22	51
5	31	31	31	90
6	33	33	33	109
7	34	34	33	68
8	33	33	33	101
9	38	38	38	91
10	36	36	36	100
11	30	30	29	60
12	34	34	33	91
13	31	31	31	66
14	49	49	49	134
15	26	26	26	93
16	32	32	32	84
17	42	42	42	84
18	23	23	23	43
19	18	18	18	62
20	15	15	15	29
21	35	35	35	85
22	34	34	34	87
23	35	35	35	75
24	35	35	35	91
25	33	33	33	71
26	24	24	25	60
27	31	31	31	95
28	23	23	23	101
29	34	34	34	245
30	32	32	32	104
31	35	35	35	134
32	32	32	32	85
33	42	42	42	123
34	8	8	8	14
35	31	31	31	79

The specificity and sensitivity of the radiofrequency SurgiSafe^®^ antenna were 100% for a total of 1,070 gauze pads, generating no false-negative and no false-positive results during the entire experiment. The manual count had a sensitivity of 99.72% and a specificity of 99.90%.

**Figure 2:**
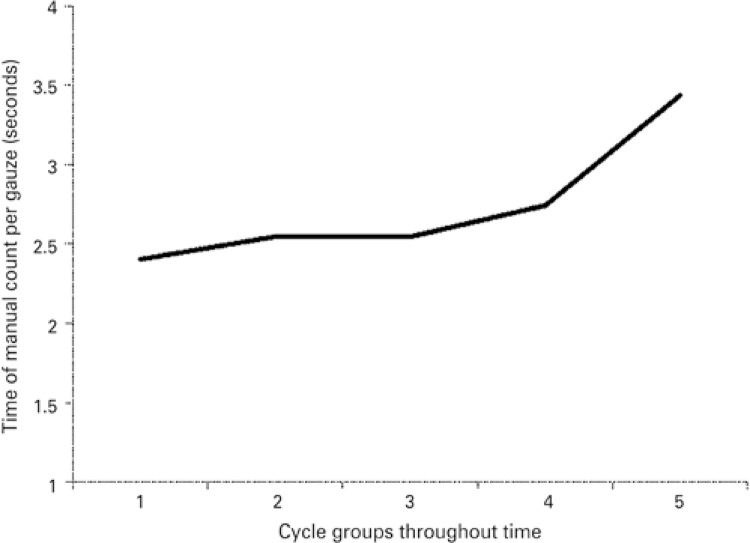
Time of manual count per gauze unit *versus* count cycles in chronological order

The manual count time varied greatly, according to the number of gauze pads, which, by randomization, ranged between 8 and 42 pads, depending on the cycle. The mean gauze pad manual counting time was 3 seconds, with a standard deviation of 1 second. SurgiSafe^®^ counting time was immediate, whereas the manual counting time tended to increase, as the cycles proceeded. We can associate this increase with fatigue of the professional (Figure 2).

Comparing manual count with the wand, no statistical difference was observed (χ^2^=20.59; p>0.98) in terms of sensitivity and specificity, and the same occurred when comparing it with the FF antenna (χ2=61.47; p>0.98). In this experiment, these two devices did not have a satisfactory performance, for their designs were not compatible with an intraoperative use, and for being professional-dependent methods, preventing a safe calculation of positive and negative predictive values.

## DISCUSSION

There was no statistical difference between SurgiSafe^®^ and manual count, but the latter differed from the correct value in four instances, in 35 repetitions. It is noteworthy that in this study, the observer's attention was focused exclusively on counting textile items, which is very different from a surgical routine. The manual count prevents 82% of IRFB^(^ ^17^ ^)^ when done correctly. In cardiac surgeries performed in New York, there are considerable discrepancies, with sensitivity of 77.2% and specificity of 99.2%.^(^ ^6^ ^)^ In 739 reports involving incomplete or missing count, 62% involved an emergency procedure.^(^ ^18^ ^)^

Regarding the time needed to count the textiles in this experiment, some facts must be considered. The fact that the pads were grouped by a thread made it much easier to identify them in the cavity, and the physical presence of TAGs facilitated the manual counting process. On the other hand, the thread made it difficult to separate gauzes. As the gauze pads became saturated and unfolded, they started to get tangled up with each other, a common occurrence during a surgery. With the fatigue of the team and the saturation of the textiles, the time needed to count each textile increased.

The literature indicates sensitivity, specificity and positive predictive value of the RF treadmill of 100% for patients with a body mass index of <40; in those with body mass index >40, a sensitivity of 96.9% was observed.^(^ ^19^ ^)^

In our experiment, the radiofrequency detection devices - wand and FF antenna - failed to demonstrate satisfactory performance. Although the RF wand had sensitivity and specificity of 100% in a study with only 8 patients, as this is an operator dependent mechanism, the potential human error in the scanning process has been recognized and reported.^(^ ^20^ ^)^

A combination of computer-aided count of tagged textiles and manual count was recently used with success.^(^ ^21^ ^,22)^ The auxiliary technology is recommended in surgical procedures, because 5% of the manual counts interpreted as correct are falsely correct.^(^ ^18^ ^)^

The organizations that implemented radiofrequency detection devices showed a 93% reduction in the incidence of IRFB in six years, as compared to a reduction of only 77% in other institutions without them.^(^ ^18^ ^)^

A cost-benefit analysis conducted by the University Health System Consortium showed that savings in X-rays, surgical time, and avoided legal medical expenses exceeded the costs involved in the use of radiofrequency technology.^(^ ^18^ ^)^

Improvements associated with manual counting and electronic counting systems should continue, since it is likely that technology, *per se*, is not infallible^(^ ^20^ ^)^ - at least for now.

## CONCLUSION

In the context of a significant incidence of cases of inadvertent retention of foreign bodies causing serious injuries to patients, with repercussions for the surgical team, hospital and health insurance providers, every possible prevention is recommended.

The benefits of the use of SurgiSafe^®^, which was effective *in vivo* in this experiment, include instant counting time and precise counting of textile items, with numerical identification of each textile item and generation of reports for hospital staff and management. Our data support the fact that the use of SurgiSafe^®^ as an aid in the manual count procedure is of great value in preventing unintended retention of foreign bodies and improving patient safety.

## References

[1] Hariharan D, Lobo DN (2013). Retained surgical sponges, needles and instruments. Ann R Coll Surg Engl..

[2] Stawicki SP, Evans DC, Cipolla J, Seamon MJ, Lukaszczyk JJ, Prosciak MP (2009). Retained surgical foreign bodies: a comprehensive review of risks and preventive strategies. Scand J Surg..

[3] Whang G, Mogel GT, Tsai J, Palmer SL (2009). Left behind: unintentionally retained surgically placed foreign bodies and how reduce their incidence–pictorial review. AJR Am J Roentgenol..

[4] Birolini DV, Rasslan S, Utiyama EM (2016). Unintentionally retained foreign bodies after surgical procedures. Analysis of 4547 cases. Rev Col Bras Cir..

[5] Stawicki SP, Moffard-Bruce SD, Ahmed HM, Anderson HL, Balija TM, Bernescu I (2013). Retained surgical items: a problem yet to be solved. J Am Coll Surg..

[6] Egorova NN, Moskowitz A, Gelijins A, Weinberg A, Curty J, Rabin-Fastman B (2008). Managing the prevention of retained surgical instruments: what is the value of counting?. Ann Surg..

[7] Birolini DV (2013). Experiência clínica de cirurgiões brasileiros com a retenção inadvertida de corpos estranhos após procedimentos operatórios.

[8] Campione BA (2009). Know the risk factors for retained foreign bodies. OR Nurse..

[9] Park CM, Choi KY, Heo SJ, Kim JS (2014). Unilateral otitis media with effusion caused by retained surgical gauze as an unintended iatrogenic complication of orthognathic surgery: case report. Br J Oral Maxillofac Surg..

[10] Moffatt-Bruce SD, Cook CH, Steinberg SM, Stawicki SP (2014). Risk factors for retained surgical items: a meta-analysis and proposed risk stratification system. J Surg Res..

[11] Iglesias AC, Salomão RM (2007). Gossipiboma intra-abdominal - análise de 15 casos. Rev Col Bras Cir..

[12] Kim KS (2014). Changes in computed tomography findings according to the chronicity of maxillary sinus gossypiboma. J Craniofac Surg..

[13] Bakan S, Kandemirli SG, Kuyumcu G, Ersen E, Tutar O (2015). Intrathoracic gossypiboma after spinal operation. Ann Thorac Surg..

[14] Velasco-Mata S, Díaz-Gómez M, Cova-Bianco T, Hopp-Mora E, Rodriguez-Rojas RR, Chirinos-Malave Y (2015). Duodenal gossypiboma: a case report and literature review. Invest Clin..

[15] Corona AR, Peniche AC (2015). A cultura de segurança do paciente na adesão ao protocolo da cirurgia segura. Rev Sobecc..

[16] Amaya MR, Maziero EC, Grittem L, Cruz EA (2015). Análise do registro e conteúdo de checklists para cirurgia segura. Esc Anna Nery..

[17] Regenbogen SE, Greenberg CC, Resch SC, Kollengode A, Cima RR, Zinner MJ (2009). Prevention of retained surgical sponges: a decision-analytic model predicting relative cost-effectiveness. Surgery..

[18] Williams TL, Tung DK, Steelman VM, Chang PK, Szekendi MK (2014). Retained surgical sponges: findings from incident reports and a cost-benefit analysis of radiofrequency technology. J Am Coll Surg..

[19] Steelman VM, Alasagheirin MH (2012). Assessment of radiofrequency device sensitivity for the detection of retained surgical sponges in patients with morbid obesity. Arch Surg..

[20] Macario A, Morris D, Morris S (2006). Initial clinical evaluation of a handheld device for detecting retained surgical gauze sponges using radiofrequency identification technology. Arch Surg..

[21] Rupp CC, Kagarise MJ, Nelson SM, Deal AM, Phillips S, Chadwick J (2012). Effectiveness of a radiofrequency detection system as an adjunct to manual counting protocols for tracking surgical sponges: a prospective trial of 2,285 patients. J Am Coll Surg..

[22] Sakorafas GH, Sampanis D, Lappas C, Papantoni E, Christodoulou S, Mastoraki A (2010). Retained surgical sponges: what the practicing clinician should know. Langenbecks Arch Surg..

